# Case report: Traumatic lumbosacral spondyloptosis with locked L5 inferior articular process

**DOI:** 10.3389/fsurg.2023.1174169

**Published:** 2023-06-26

**Authors:** Tao Tang, Yuchi Liu, Jian Cao, Tianlong Wu, Dingwen He, Xigao Cheng, Shuihua Xie

**Affiliations:** ^1^ The First Department of Orthopedics, Jiangxi Province Hospital of Integrated Chinese and Western Medicine, Nanchang, China; ^2^ Department of Orthopedics, The Second Affiliated Hospital of Nanchang University, Nanchang, China; ^3^ Institute of Orthopaedics of Jiangxi Province, The Second Affiliated Hospital of Nanchang University, Nanchang, China; ^4^ Institute of Minimally Invasive Orthopaedics of Nanchang University, The Second Affiliated Hospital of Nanchang University, Nanchang, China

**Keywords:** traumatic lumbosacral spondyloptosis, lumbosacral dislocation, reduction, posterior lumbar fusion, case report

## Abstract

**Background:**

Traumatic lumbosacral spondyloptosis is a very rare spinal disease caused by high-energy trauma. We report a case of traumatic lumbosacral spondyloptosis with locked L5 inferior articular process.

**Case presentation:**

A 33-year-old man presented with multisite pain for 6 h following waist trauma and was admitted to the hospital. He suffered multiple injuries from severe impact on the waist after driving an out of control forklift truck. Preoperative imaging examinations revealed that the patient was diagnosed with traumatic lumbosacral spondyloptosis and the L5 inferior articular process was locked into the anterior margin of the S1 vertebra. A posterior instrumentation, decompression of the cauda equina, and interbody fusion procedure was performed. The patient received hyperbaric oxygen and rehabilitation treatment 10 days after the surgery. At the 6-month postoperative follow-up, the muscle strength of the lower limbs was improved, the patient had no numbness of both lower limbs, and the urinary retention symptom was significantly improved. The American Spinal Injury Association grade improved from grade C preoperatively to grade D postoperatively. As far as we know, there have been no relevant reports on traumatic lumbosacral spondyloptosis with locked L5 inferior articular process yet.

**Conclusion:**

We believe that the hyperflexion and shear forces were the potential causes of this injury. In addition, the preoperative imaging examinations should be evaluated carefully. If the inferior articular process of L5 were locked, we suggest removing the bilateral inferior articular processes first and then perform reduction.

## Introduction

Traumatic lumbosacral spondyloptosis is a very rare spinal disease caused by high-energy trauma and it is defined as 100% or greater displacement of S1 vertebral body over the L5 vertebral body in the coronal or sagittal plane to an injury; the majority of cases will lead to serious cauda equina injuries and dural tear (1–4). Although traumatic lumbosacral spondyloptosis cases are rarely described in clinical practice, this injury still needs to be emphasized in consideration of the severe clinical manifestations and poor prognosis due to the complete loss of spinal column alignment (2). According to the Meyerding classification, traumatic lumbosacral spondyloptosis is described as grade V spondylolisthesis, in which imaging findings showed a subluxation of >100% of adjacent vertebral bodies (4). Once diagnosed, surgical treatment should be performed as soon as possible if there is no obvious contraindication for surgical intervention (5, 6). Surgical treatment is used to restore spinal balance, weight-bearing ability, and neurological function for early mobilization of the patients (6, 7). The combination of posterior instrumentation, decompression of the cauda equina, and interbody fusion is the main surgical method for the treatment of traumatic lumbosacral spondyloptosis (2, 3). As far as we know, there have been no relevant reports on traumatic lumbosacral spondyloptosis with locked L5 inferior articular process yet. In this paper, we report a rare severe case of traumatic lumbosacral spondyloptosis with locked L5 inferior articular process, and some operative techniques for reduction of the L5 vertebra body were introduced.

## Case presentation

### Clinical history and imaging findings

**Figure 1:**
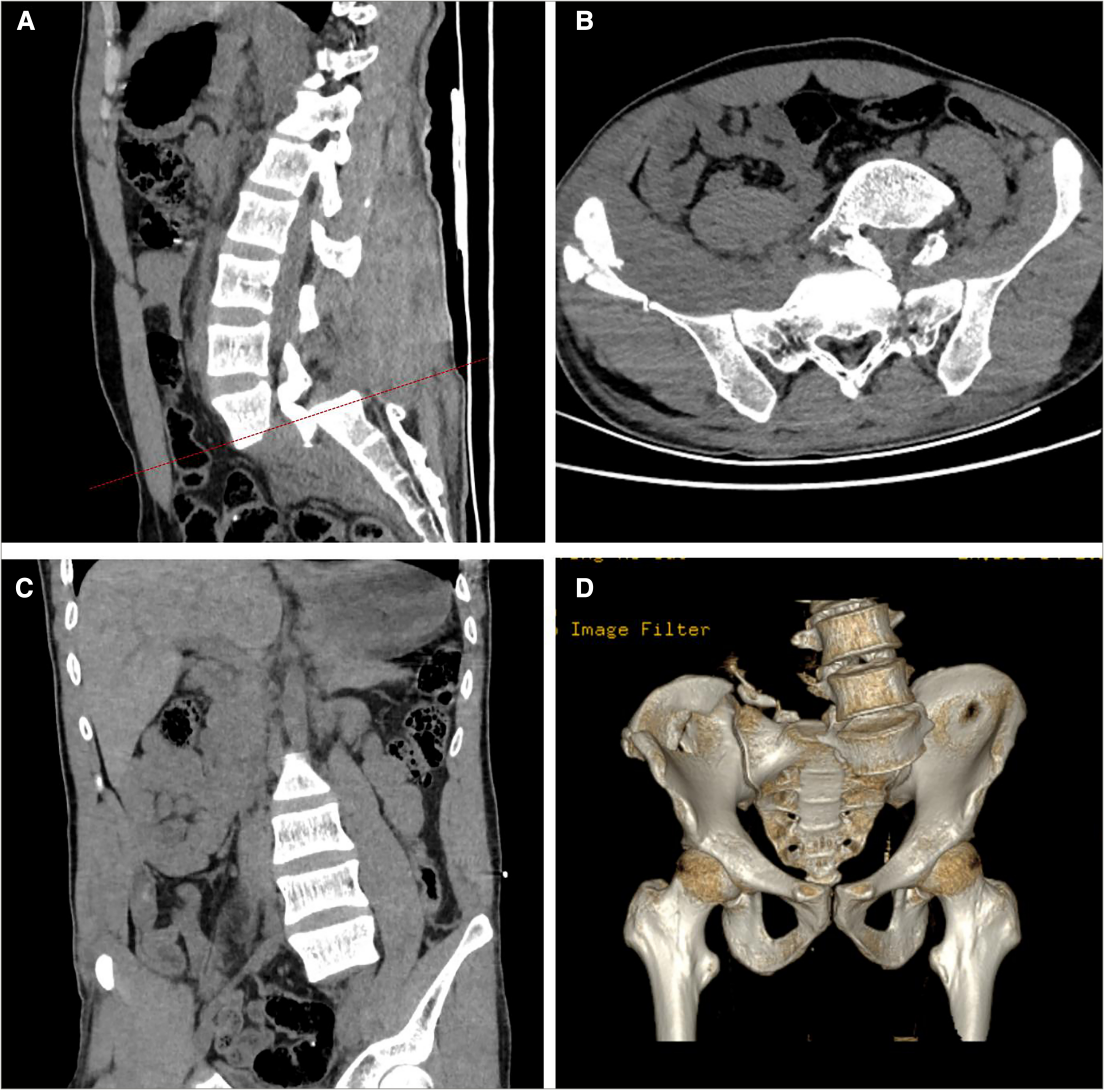
Sagittal (**A**), cross-sectional (**B**), and coronal (**C**) CT images showed the patient was definitely diagnosed with lumbosacral spondyloptosis. The three-dimensional reconstruction was useful in showing the bony structure clearly (**D**). CT, computed tomography.

**Figure 2:**
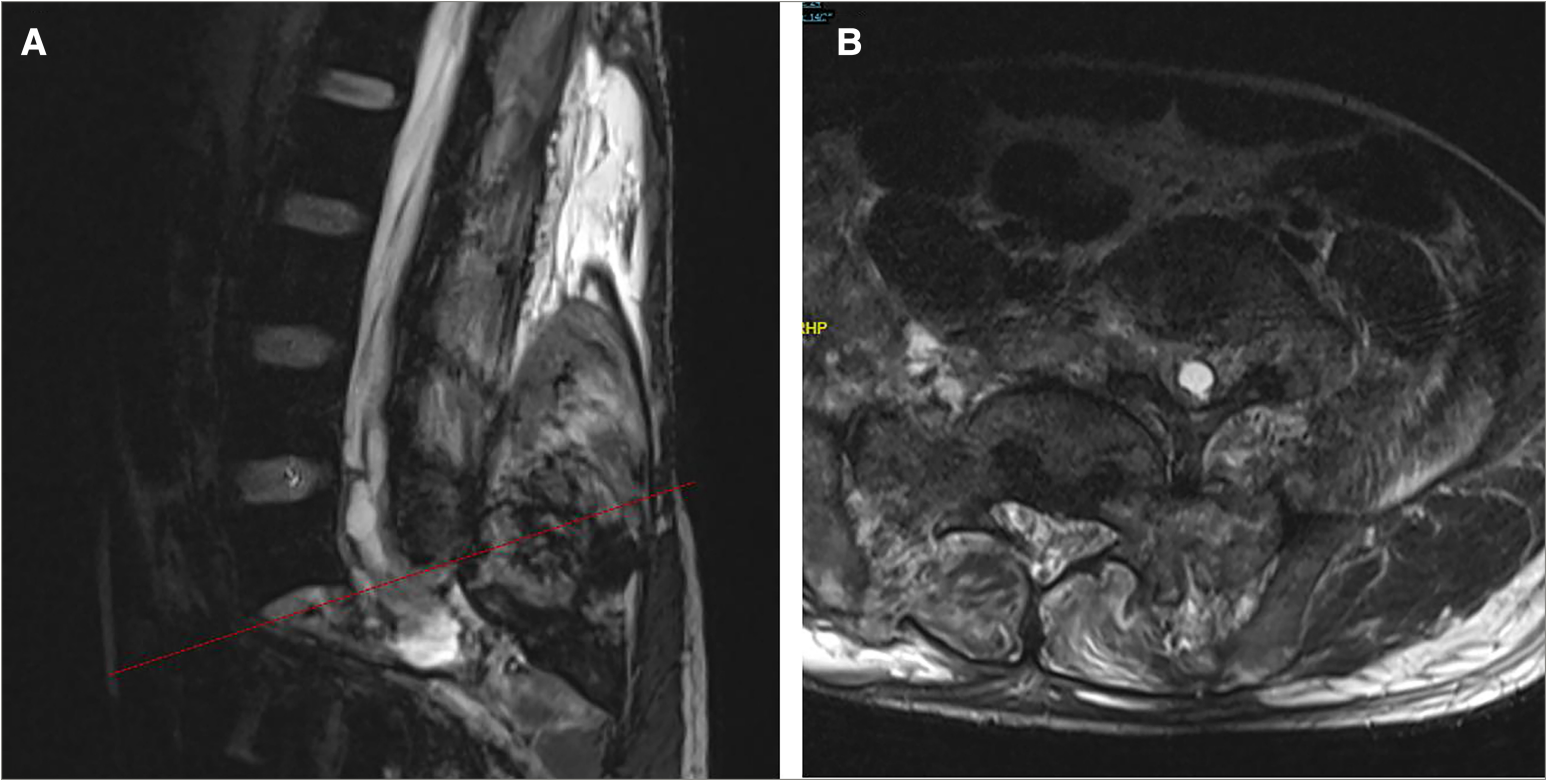
Sagittal (**A**) and cross-sectional (**B**) MRI images showed the dural tear and cerebrospinal fluid leakage. MRI, magnetic resonance imaging.

A 33-year-old man complained of multisite pain for 6 h following waist trauma and was admitted to the hospital. He suffered multiple injuries from severe impact on the right rear of his waist after driving an out of control forklift truck. The patient presented with excruciating pain in the back, chest, and abdomen, and the muscle strength of both lower limbs was decreased (3/5 strength in the left lower limb and 2/5 strength in the right lower limb); both lower limb extremity paresthesias and urinary retention was observed. Immediately after the injury, the patient was sent to our hospital by ambulance for treatment and underwent x-ray and computed tomography (CT) scan (Figure 1); the imaging examinations revealed the left 10th and 11th rib, bilateral transverse processes (L2–L5 levels), sacrum, and coccyx and right ilium fractures, and L5 vertebra and S1 vertebra dislocation and separation. A lumbosacral vertebra magnetic resonance imaging (MRI) revealed a complete rupture of the L5–S1 intervertebral disc; tear in anterior longitudinal ligament, posterior longitudinal ligament, ligamentum flavum, interspinous ligament, supraspinous ligament, and dural sac; and cerebrospinal fluid (CSF) seepage into the tissue space behind the lumbar spine (Figure 2). The patient was healthy previously and had no other diseases or syndromes. Traumatic lumbosacral spondyloptosis diagnosis was established. On admission, the patient was conscious, the blood pressure (BP) was 85/60 mmHg, and heart rate was 78 beats/min; intravenous rehydration therapy was immediately started because of a low BP and then the BP gradually returned to the normal level. The severity of the spinal injury was graded according to the American Spinal Injury Association (ASIA) grading scale, and the patient was classified as grade C. The visual analog scale (VAS) score was 9.

### Operative procedures

**Figure 3:**
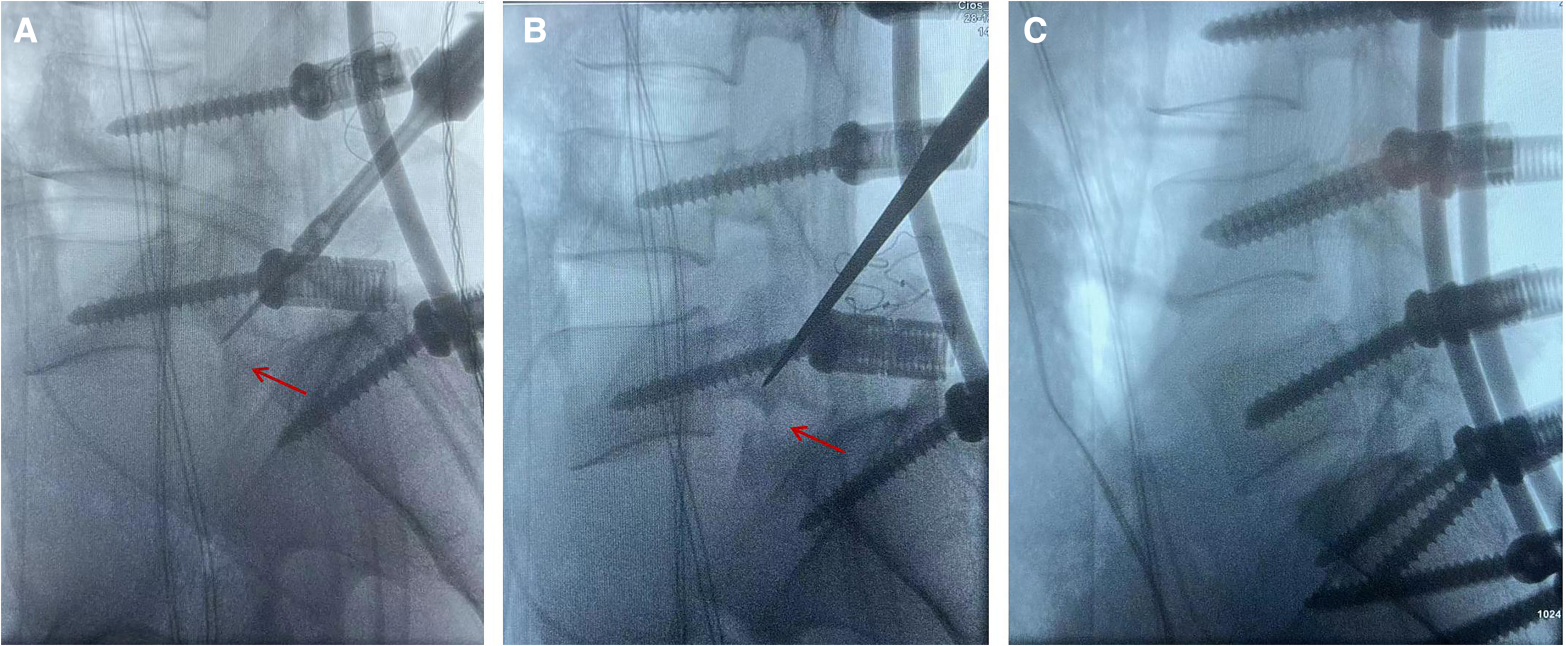
Intraoperative C-arm fluoroscopy revealed that the L5 inferior articular process was locked into the anterior margin of the S1 vertebra (**A**). Intraoperative C-arm fluoroscopy suggested that the L5 inferior articular process has been removed (**B**). Intraoperative C-arm fluoroscopy showed a good reduction (**C**).

The patient was sent to the operating room for surgical treatment 10 days after admission. During the operation, we found that the subcutaneous soft tissue and muscle behind the lumbar spine were seriously injured, the L5 vertebra were severely dislocated to the front-left side, the L5–S1 facet joint was fractured and dislocated, the bilateral inferior articular processes of L5 were locked and located anterior to the S1 vertebra (Figure 3A), severe dural sac tear had occurred at the L5–S1 level, and only part of cauda equina and filum terminale are continuous; the L5–S1 intervertebral disc had ruptured and prolapsed into the spinal canal. Specific procedures were performed as follows. After successful anesthesia, the patient was placed in the prone position. In the surgical area, routine disinfection was carried out and laid with sterile sheets. A 20–25 cm incision was made at the midline of the back using the L4 spinous process as the center. The skin was cut parallel to the spine, then the lumbodorsal fascia was stripped and the paravertebral muscle was bluntly stripped to completely expose the spinous process and bilateral vertebral plate of L3–S2 vertebrae, and the spinous process of L5–S1 vertebrae was removed. Pilot holes for pedicle screws placement from L3 through S1 and also for the S2 alar-iliac (S2AI) screws entry site were marked; bilateral L3, L4, L5, and S1 pedicle screws of appropriate length were then inserted, and dual S2AI screws placement bilaterally were performed. A pre-bent short titanium rod was temporarily installed to distract L5–S1 intervertebral space. We found that the bilateral inferior articular processes of L5 was still dislocated in front of the S1 vertebra after C-arm fluoroscopy, and it is particularly difficult to perform the reduction with a screw distractor; so we decided to resect bilateral inferior articular processes of L5 with the assistance of the ultrasonic bone scalpel (Figure 3B). Reduction was attempted again, and the spinal column getting back into normal alignment was observed after C-arm fluoroscopy (Figure 3C). Then, bilateral laminectomies, facetectomies, decompression of the cauda equina, and discectomy of L5–S1 were performed. Interbody bone graft fusion was performed by implanting an interbody fusion cage filled with bone morphogenetic protein (BMP) and local bone at the L5/S1 level. Finally, the dura with artificial dura mater were repaired. Bilateral lumbar drainage tubes were placed at the end of procedures. The operative procedure was about 3 h and intraoperative blood loss was about 800 ml.

### Postoperative course

**Figure 4:**
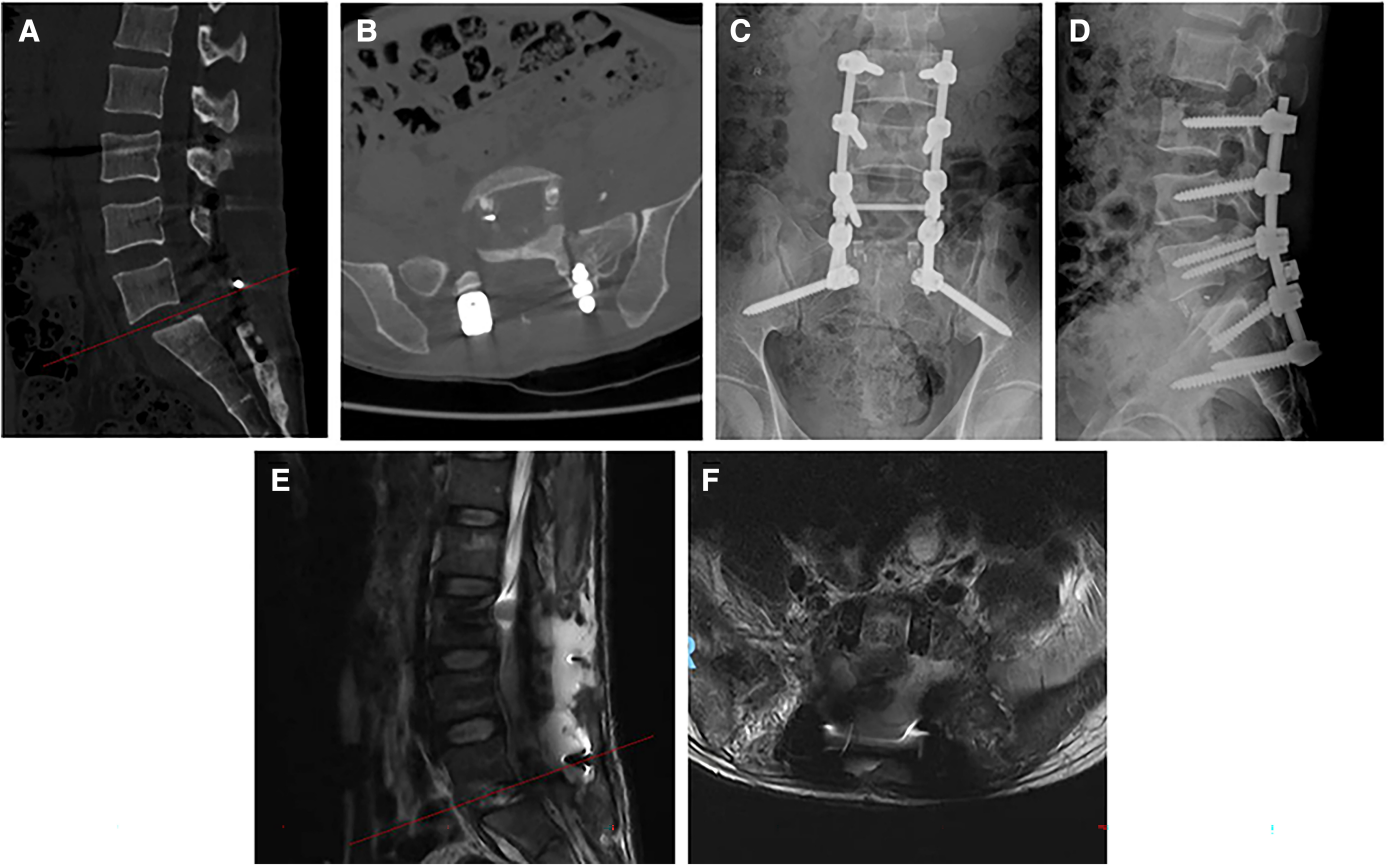
Sagittal and cross-sectional CT images at the postoperative month 1 (**A,B**). Lumbar anteroposterior and lateral x-ray radiographs at the postoperative month 1 (**C,D**). Sagittal and cross-sectional MRI images at the postoperative month 1 (**E,F**). CT, computed tomography; MRI, magnetic resonance imaging.

After the surgical operation was completed, the patient was transferred to the postanesthesia care unit and then to the orthopedics wards. We found the patient had CSF leakage on the first postoperative day and presented with mild headaches and light-headedness. The patient's low back pain and numbness of both lower limbs were effectively improved; however, the muscle strength of both lower limbs did not significantly improve. The VAS back pain decreased from 9 preoperatively to 4 postoperatively. The ASIA grade remained in grade C after surgery. The right lumbar drainage tube and the Foley catheter were removed on the third postoperative day, and then the left lumbar drainage tube was removed and the skin was closed using 2–0 nylon sutures on postoperative day 7. The patient was then transferred to the rehabilitation hospital for hyperbaric oxygen and rehabilitation treatment on postoperative day 10. At 1-month follow-up, the patient's muscle strength of bilateral hip flexors and knee extensors improved gradually, but the muscle strength of right foot dorsiflexion and plantar flexion did not show significant improvement. The lumbar x-ray, CT scan, and MRI showed that the internal fixation and lumbar interbody cages were in good position, and there was a small amount of fluid behind the lumbar spine (Figure 4). At the 6-month postoperative follow-up, the patient had no numbness of both lower limbs, and the symptom of urinary retention was significantly improved. The patient exhibited 4/5 strength in the left lower limb, 2/5 strength in right foot dorsiflexion and plantar flexion, and 4/5 strength in right hip flexor and knee extensor. The ASIA grade improved from grade C preoperatively to grade D postoperatively.

## Discussion and conclusions

Lumbosacral spondyloptosis is a special type of lumbosacral dislocation; although there has been no study to explain the biomechanical mechanism of traumatic spondyloptosis, some literature studies on biomechanical mechanism of lumbosacral dislocation have been reported. The lumbosacral dislocation was first described by Watson-Jones in 1940, and the mechanism of injury was considered to be correlated with the high hyperextension forces (8). In recent years, more and more studies were reported and our understanding of the biomechanics of this injury was improved. Some scholars believe that a combination of compression, translation, hyperextension, and rotational forces was the cause of traumatic lumbosacral dislocation (9), whereas others believe that the vectors of hyperflexion with distraction and shear forces were the potential causes of this injury (10). In our case, the patient suffered a violent impact on the right rear of the waist, and this led to severe hyperflexion at the waist and led to the bilateral inferior articular processes of L5 dislocation to the front of the S1 vertebra. Therefore, the injury mechanism of the latter seems more reasonable.

Due to the complexity of this injury, some scholars classify it according to its anatomical structure. Aihara et al. (11) classified the injury into five types. Type 1 is unilateral facet dislocation with or without facet fracture, type 2 is bilateral facet dislocation with or without facet fracture, type 3 is unilateral facet dislocation and fracture of the contralateral facet, type 4 is dislocation of the L5 vertebra body with bilateral fracture of the pars interarticularis, and type 5 is dislocation of the L5 vertebra body by a body fracture or the pedicle with or without injury of the lamina or facets. In 2007, Vialle et al. (12) proposed a more detailed classification method based on the injury of articular process and intervertebral disc. In our case, a bilateral facet fracture with rotational displacement associated with complete dislocation of L5 was found via MRI and CT scan; therefore, the patient belongs to type IIIB (13–15). According to the AOSpine Classification, lumbosacral spondyloptosis is categorized as a type C with severe biomechanical instability that requires surgical treatment.

Traumatic lumbosacral spondyloptosis can lead to serious spinal instability and sagittal or coronal dislocation, which can be clearly diagnosed by three-dimensional CT reconstruction; it is usually accompanied by severe neurologic impairment, dural tear, and other injuries (2). Murata et al. (15) described a case of partial avulsion of cauda equina. The biomechanics is explained by traction forces on the cauda equina due to the dislocation of L5 vertebral body. In 2016, Adelved et al. (16) reported that the rate of neurologic deficits corresponding to L5 and sacral roots in patients with lumbosacral dislocation was 84.6% (11/13). Some reports suggested that the time of decompression, the grade of slip, and the degree of neurologic deficits before operation are the main factors affecting the recovery of neurologic function (2, 4, 17).

In a focused review, Vialle et al. (12) proposed that surgical treatment should be performed for all types of lumbosacral dislocation. At present, the main surgical treatment is posterior instrumentation, decompression of the cauda equina, and interbody fusion; the anatomical reduction of L5 vertebra and repair of dural sac are very important, and reliable fusion of the vertebrae is crucial to the stabilization of the spine after surgery. If the bone quality is poor and spondylolisthesis is with higher grades, the fixed segment can be appropriately extended up or down. In our case, the patient was grade V according to the Meyerding classification. We choose the fixed segment L3–S2 to achieve the ideal anatomic reduction, and the bilateral S2AI screws were placed by the free hand technique. However, the right inferior articular process of L5 locked into the anterior margin of the S1 vertebra was observed intraoperatively. Therefore, we chose to properly distract the L5–S1 intervertebral space after pedicle screw placement and gradually expose the bilateral inferior articular processes and isthmus of L5; then, the bilateral inferior articular processes of L5 were excised with the assistance of an ultrasonic bone scalpel. Next, we performed reduction again and achieved great results. However, as a single case report, this study has several limitations. It is necessary to evaluate additional cases to validate these findings; in addition, further research is needed to establish the effectiveness of the described surgical approach for other patients with similar injuries.

In conclusion, traumatic lumbosacral spondyloptosis is a rare ailment and usually the result of high-energy trauma. Early spinal surgery and restoration of normal alignment should be the superior choice. In addition, preoperative imaging examinations should be evaluated carefully. If the inferior articular process of L5 were locked, we suggest removing the bilateral inferior articular processes first and then perform reduction.

## Data Availability

The original contributions presented in the study are included in the article, further inquiries can be directed to the corresponding author.
